# Polymorphism of *FABP2* and *PPARG2* genes in risk prediction of cataract among North Indian population^☆^

**DOI:** 10.1016/j.mgene.2014.02.002

**Published:** 2014-04-17

**Authors:** Shania Abbas, Syed Tasleem Raza, Anu Chandra, Luxmi Singh, Saliha Rizvi, Ale eba, Faisal Ahmed, Farzana Mahdi

**Affiliations:** a Department of Biochemistry, Era's Lucknow Medical College and Hospital, Lucknow 226003, India; b Department of Ophthalmology, Era's Lucknow Medical College and Hospital, Lucknow 226003, India

**Keywords:** *FABP2*, *PPARG2*, Cataract, Genetic polymorphism

## Abstract

**Background:**

Cataract is the leading cause of bilateral blindness in India. It has been reported that cataract is responsible for 50–80% of the bilaterally blind in the country. Cataract formation is a natural part of the ageing process. At present, adequate data are not available regarding the *FABP2* and *PPARG2* gene polymorphisms and their susceptibility with cataract cases in the North Indian population. Thus, the present study was carried out to investigate the association of *FABP2* and *PPARG2* gene polymorphisms with cataract cases and controls.

**Materials and methods:**

This study includes 130 cataract cases and 118 controls. *FABP2* and *PPARG2* gene polymorphisms in cases and controls were evaluated by PCR-RFLP.

**Results:**

Frequencies of Ala54Ala, Ala54Thr and Thr54Thr genotypes in *FABP2* gene in cataract cases and controls were 50.76%, 39.23%, 10% and 25.42%, 61.86%, 12.71% respectively. The *PPARG2* gene CC, CG, GG genotype frequencies were 11.53%, 87.69% and 0.76% in cases and 21.18%, 39.83% and 38.98% in healthy controls respectively. Significant differences were observed in the frequencies of *FABP2* Ala54Ala, Ala54Thr genotype (p < 0.05) and *PPARG2* CC, CG, GG genotype (p < 0.05) between cases and controls.

**Conclusion:**

The findings of this study suggest that *FABP2* and *PPARG2* gene polymorphisms can be an informative marker for early identification of population at risk of cataract. The potential role of *FABP2* and *PPARG2* gene polymorphisms as a marker of susceptibility to cataract needs further studies in a larger number of patients.

## Highlights

• The frequency of FABP2 Ala54Ala genotype was significantly higher in cases.
• The frequency of Ala54Thr genotype was significantly lower in cases.
• The frequency of PPARγ2 CC and GG genotype was considerably lower in cases.

## Introduction

Cataract is a clouding that develops in the crystalline lens of the eye or in its envelope varying in degree from slight to complete opacity and obstructing the passage of light. Age related cataracts are responsible for 48% of world blindness, which represents about 18 million people, according to the World Health Organization (WHO) (Dua et al., 2009). It is a multifactorial disease which is associated with many environmental (McCarty and Taylor, 2002) and genetic variations (Hejtmancik and Kantorow, 2004, McCarty and Taylor, 2001, Shiels and Hejtmancik, 2007). Epidemiologic studies have shown that cataract is associated with many environmental factors such as ultraviolet B light exposure, smoking, alcohol consumption and use of steroids (Hiratsuka and Li, 2001, Klein et al., 2001, McCarty and Taylor, 2002, Tan et al., 2008, Wang et al., 2008). Recently, genetic factors have been found to play important roles in the development of senile cataract (Hejtmancik and Kantorow, 2004, McCarty and Taylor, 2001, Shiels and Hejtmancik, 2007).

The intestinal fatty acid-binding protein-2 (*FABP2*) gene codes a protein expressed in enterocytes and is responsible for the absorption of long-chain fatty acids (Weiss et al., 2002a, Weiss et al., 2002b). The gene for *FABP*2 is located in the long arm of chromosome 4. The G to A polymorphism (rs1799883) of codon 54 results in the substitution of threonine (Thr) for alanine (Ala) (Baier et al., 1995). A single nucleotide polymorphism (SNP) in the *FABP2* gene at codon 54 causes an amino acid change (Ala to Thr). Carriers of the Thr54 allele in *FABP2* gene have a twofold greater affinity for the absorption of long-chain fatty acids than those with the Ala54 allele (Agren et al., 2001), which supports the role of the *FABP*2 Ala54Thr polymorphism in the etiology of obesity and metabolic disorders. Thr54 allele is significantly associated with higher total cholesterol, stroke incidence (Carlsson et al., 2000), elevation of fasting and postprandial triglyceride (Georgopoulos et al., 2000), insulin resistance (Baier et al., 1995, Weiss et al., 2002a, Weiss et al., 2002b), and higher non-esterified fatty acid concentrations (Pratley et al., 2000).

Peroxisome proliferator-activated receptors (PPARs) are ligand-activated transcription factors in the nuclear hormone receptor superfamily related to retinoid, steroid and thyroid hormone receptors (Abdelrahman et al., 2005). The PPAR family is represented by three members: *PPARα*, *PPARβ/δ*, and *PPARγ* (Tyagi et al., 2011). The *PPARγ* gene contains 9 exons which spans more than 100 kb, and because of alternative mRNA splicing it results in the production of 2 protein isoforms: *PPARγ1* and *PPARγ2* (Fajas et al., 1997). The anti-inflammatory effects of *PPARγ* have been observed in various organs, although previous investigations mainly focused on internal organs, such as the kidneys (Taguchi et al., 2012), heart (Yu et al., 2012), and lungs (Reddy et al., 2012). In addition, a *PPARγ* agonist inhibited fibrotic changes by suppressing transforming growth factor beta (TGF-β) signaling (Hatanaka et al., 2012). In the present study, we examined the association between *FABP2* and *PPARG2* gene polymorphisms in cataract cases among North Indian population.

## Materials and methods

### Patient's selection

A total of 130 blood samples of cataract cases and 118 healthy controls were collected from the Department of Ophthalmology of Era's Lucknow Medical College & Hospital, Lucknow. Data collection was done for each patient on clinical variables including age, alcohol consumption, body mass index, height, weight, cigarette smoking, family history etc. All subjects with senile cataract (72 males, 59 females) had visual disturbance and their corrected visual acuities were under 6/24. We excluded patients with secondary cataract due to diabetes, trauma, steroid administration, and other causes. The age-matched control subjects were collected from unrelated volunteers in the same clinic. Informed consent was obtained from each subject before the study. Ethical committee's (institutional ethics committee no. ELMC/E-1/2010/3942) clearances were obtained from the respective departments earlier before the recruitment of subjects in this study.

### DNA extraction

Five milliliters of peripheral blood was collected from all the subjects in 0.5 M EDTA tubes. Genomic DNA was isolated from whole blood using the standard phenol–chloroform extraction method (Sambrook et al., 1989). The DNA concentration was determined by spectrophotometer and stored at − 20 °C.

## Analysis of polymorphisms

### FABP2 polymorphism

PCR was employed for genotyping of the *FABP2* gene polymorphism. Reactions were performed using 10 pmol of each primer: forward primers 5′-ACAGGTGTTAATATAGTGAAAAG-3′ and reverse primer 5′-TACCCTGAGTTCAGTTCCGTC-3′. The final volume of PCR (MJ Mini Thermo Cycler-BioRad) reaction mixture was 20 μl containing 200 ng of genomic DNA, 0.3 U of Taq DNA polymerase (Bioline Ltd., London, UK), 10 mmol/l Tris–HCl pH 8.3, 50 mmol/l of KCl, 1.5 mmol/l of MgCl_2_, 100 mmol/l of dNTPs and 10 pmol of each primer. PCR amplification was carried out under the conditions: 35 cycles for 1 min at 94 °C, 1 min at 5 °C and 1 min at 72 °C. The PCR products thus obtained were analyzed on 2% agarose gel stained with ethidium bromide to certify the proper amplification. The amplified PCR products of 180 bp were digested with the addition of 2 U HhaI (New England Biolabs), 10 mmol/l Tris–HCl pH 7.9, 50 mmol/l NaCl, 10 mmol/l MgCl_2_ and 1 mmol/l dithiothreitol. After incubation at 37 °C for 2 h with restriction enzyme HhaI, digested samples were separated by polyacrylamide gel electrophoresis on 10% ethidium bromide stained polyacrylamide gel and visualized by UVP BIOLMAGING gel doc system. PCR products having an intact HhaI site were cleaved into 99 and 81 bp fragments; the Ala54Thr substitution abolished the restriction site (Fig. 2).

### PPARG2 polymorphism

**Fig. 1:**
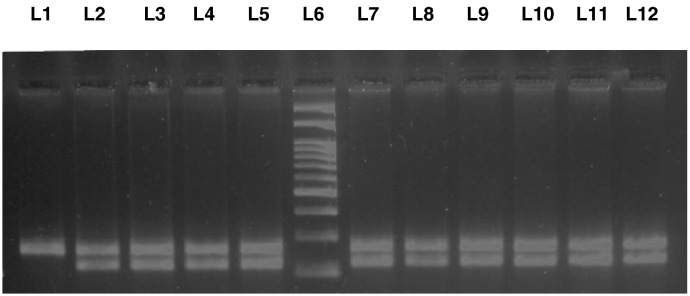
Agarose gel picture showing PCR-RFLP product of PPARγ2 gene, lane 1 shows undigested product, lanes 2, 3, 4, 5, 7, 8, 9, 10, 11, and 12 show CG(+/-) genotype and lane 6 shows 100 bp ladder.

**Fig. 2:**
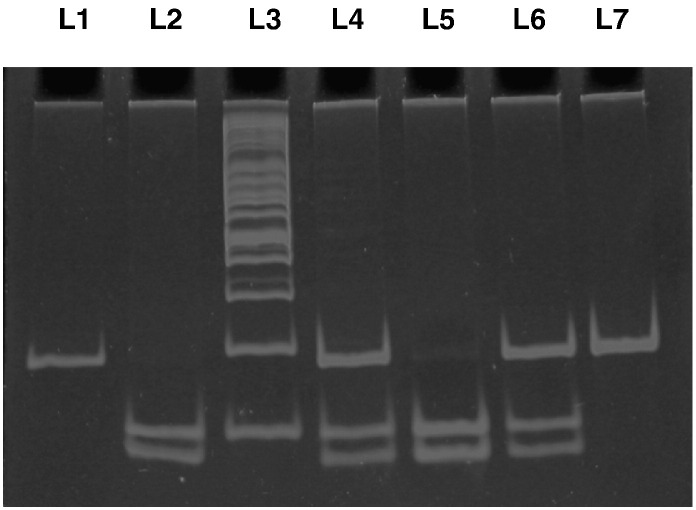
Polyacrylamide gel picture showing digested PCR products for FABP2 gene polymorphism. L1: undigested PCR product of FABP2 (180 bp), L2 and L5: TT genotype, L4 and L6: AT genotype, L7: AA genotype, L3: 100 bp ladder.

Genotyping was performed using PCR-RFLP (MJ Mini Thermo Cycler-BioRad). with the following primers: forward primer 5′-CAA GCC CAG TCC TTT CTG TG-3′ and reverse primer 5′-AGT GAA GGA ATC GCT TTC CG-3′ (Ek et al., 1999). All reactions were performed in a total volume of 50 μl containing 10 mmol/l Tris–HCl (pH 8.8), 50 mmol/l KCl, 1.5 mmol/l MgCl_2_, 0.2 mmol of each dNTPs, 50 pmol of each of the two primers, 0.3 U of Taq DNA polymerase (Bioline Ltd., London, UK) and 200 ng of genomic DNA. PCR amplification was carried out under the conditions: initial precycling denaturation by holding at 94 °C for 3 min, denaturation for 40 cycles at 94 °C for 30 s followed by annealing at 53 °C for 30 s, extension at 72 °C for 1 min and a final extension period at 72 °C for 9 min. The PCR products were incubated over night with 2 U of the restriction enzyme Hpa II (New England Biolabs, Hitchin, UK). This cuts the mutant allele at a site introduced by the reverse primer. The digested samples were applied to 3% agarose gel, subjected to electrophoresis for about half an hour and visualized on a UV transilluminator (Fig. 1). *PPARG2* homozygous for CC genotype corresponded to the 106-bp fragment, whereas the heterozygous CG was characterized by two fragments of 106 and 130 bp (Fig. 2).

### Statistical analysis

All the figures are presented as means ± SD. The genotyping data were compared between cases and controls using Chi-square test. Other variables were compared using Student's t-test for normally-distributed variables. All statistical tests were performed using SPSS (Statistical Package for the Social Sciences) version 12 software.

## Results

Our study included 131 cataract cases (72 were males and 58 were females) and 126 controls (64 were males and 54 were females). Mean age of the cases in this study was 53.74 ± 11.87 years, while in control group it was 52.02 ± 12.11 years. Clinical and biochemical parameters of cases and controls are shown in Table 1. Frequencies of *FABP2* Ala54Ala, Ala54Thr and Thr54Thr in cataract cases and controls were 50.76%, 39.23%, 10% and 25.42%, 61.86%, 12.71% respectively. The Odds Ratio (OR) for Ala54Ala was 3.03 (95% CI 1.76–5.18, p < 0.0001, χ2 = 16.75, power = 0.97); for Ala54Thr, 0.40 (95% CI 0.24–0.66, p = 0.0004, χ2 = 12.67, power = 0.923) and for Thr54Thr, 0.76 (95% CI 0.35–1.68, p = 0.500, χ2 = 0.45, power = 0.549). The frequencies of Ala and Thr alleles in cases were 70.38% and 29.61% as compared to 56.35% and 43.64% in the controls. Odds Ratio for Ala was 1.84 (95% CI 1.27–2.67, P = 0.001, χ2 = 10.53, power = 0.922) and for Thr it was 0.54 (95% CI 0.38–0.79, P = 0.001, χ2 = 10.53, power = 0.922). The *PPARG2* gene CC, CG, GG genotype frequencies were 11.53%, 87.69% and 0.76% in cases and 21.18%, 39.83% and 38.98% in healthy controls respectively. OR for CC was 0.49 (95% CI 0.24–0.97, p = 0.039, χ2 = 4.26, power = 0.765); for CG, 10.76 (95% CI 5.68–20.41, p < 0.0001, χ2 = 62.22, power = 0.999) and for GG, 0.01 (95% CI 0.002–0.090, p < 0.0001, χ2 = 58.81, power = 0.999). The frequencies of C and G alleles in cataract cases were 55.38% and 44.61% as compared to 41.10% and 58.89% in the controls. Odds Ratio for C was 1.79 (95% CI 1.25–2.54, P = 0.001, χ2 = 10.10, power = 0.919) and for G, 0.56 (95% CI 0.39–0.80, P = 0.001, χ2 = 10.10, power = 0.919). The genotype and allele frequencies of *FABP2*, *PPARG2* genes and the statistical analysis among the cases and controls are shown in Table 2.

## Discussion

Cataracts are a clinically and genetically heterogeneous group of eye disorders that causes visual impairment. At least 34 loci and mutations in 22 genes have been reported to be linked with different forms of cataracts. Genetic factors are considered the most important factors in the development of senile cataract. Previously, many studies have investigated the association between genetic polymorphisms and cataract.

### FABP2 gene polymorphism

The *FABP2* gene has been proposed as a candidate gene for diabetes and insulin resistance because the protein it encodes is involved in fatty acid absorption and metabolism (Weiss et al., 2002a, Weiss et al., 2002b). Among Pima Indians, who are known to have the highest prevalence of type 2 diabetes mellitus (T2DM), the Thr encoding allele (Thr54) is associated with insulin resistance and enhanced fat oxidation rates (Baier et al., 1996, Knowler et al., 1990). The intestinal fatty acid-binding protein (FABP2) gene located at chromosome 4q28-31 is a candidate gene possibly implicated in insulin resistance and the pathogenesis of type 2 diabetes mellitus. Several studies have reported associations between this polymorphism and insulin resistance, dyslipidemia, stroke, metabolic syndromes and hypertriglyceridemia (Carlsson et al., 2000, Vimaleswaran et al., 2006, Weiss et al., 2002a, Weiss et al., 2002b). The frequency of *FABP2* Ala54Ala genotype was 50.76% in cases which is significantly higher in comparison with control 25.42% (p < 0.0001). The frequency of Ala54Thr genotype in our study was 39.23% in cases which is significantly lower in comparison with controls where it was 61.86% (p = 0.0004).

### PPARG2 gene polymorphism

The association between the substitution of G (alanine) for C (proline) at codon 12 of *PPARG2* gene and the risk for type 2 diabetes mellitus has been widely studied since Yen first reported this polymorphism (Yen et al., 1997). Cataracts are more common in people with diabetes than in the general population under the age of 40 years and they are morphologically similar to senile cataracts. The exact correlation between cataracts and T2DM is without a definite conclusion. Cataracts are a significant complication of T2DM (Liu et al., 2009, McDonough et al., 2009, Unoki et al., 2008). Most have found that the carrier of the G (Ala 12) allele had a lower risk for T2DM and insulin resistance (Deeb et al., 1998). In our study we have reported that the frequency CG genotype (Pro12Ala) was 87.69% in cases which is significantly higher in comparison with the controls 39.83% (p < 0.0001). The frequency of *PPARG*2 CC and GG genotypes in our study group was 11.53%, 0.76% in cases which is considerably lower in comparison to the controls where it was 21.18% and 38.98% (p = 0.039, p < 0.0001), respectively. According to our data, the frequency of C allele in cases was 55.38% which is significantly higher than the controls 41.10% (p = 0.001).

## Conclusion

The findings of this study suggest that *FABP2* and *PPARG2* gene polymorphisms can be an informative marker for the early identification of population at risk of cataract. The potential role of *FABP2* and *PPARG2* gene polymorphisms as a marker of susceptibility to cataract needs further studies in a larger number of patients.

## Figures and Tables

**Table 1:** Clinical and biochemical parameters of cataract cases and controls.

Parameters	Cataract cases (n = 130)	Control (n = 118)
Gender (M/F)	72/58	64/54
Age (yrs)	52.02 ± 12.11	53.74 ± 11.87
Wt. (kg.)	51.98 ± 10.43	57.57 ± 11.39
SBP (mm Hg)	126.04 ± 16.92	122.30 ± 2019
DBP (mm Hg)	81.82 ± 8.83	82.15 ± 19.30
RBS (mg/dl)	129.31 ± 30.35	124 ± 61.47
Hb (gm%)	11.65 ± 1.19	14.45 ± 1.65
TLC (cell/cumm)	7792 ± 2029	7571.70 ± 1690.06
Neutrophils (%)	65.51 ± 9.35	65.35 ± 6.73
Lymphocytes (%)	29.14 ± 8.00	28.70 ± 6.73
Eosinophils (%)	4.83 ± 3.94	4.58 ± 2.89
Monocytes (%)	2.47 ± 13.81	1.19 ± 3.20
Pl. count. (Lakh/cumm)	2.22 ± 0.77	1.99 ± 0.68

n = number of subjects.

**Table 2:** Genotype & allele frequencies of *FABP2* & *PPARG2* genes in cataract cases and healthy controls. Bold values indicate p value is less than 0.05.

FABP2						
Group	Alleles & genotype	AA	AT	TT	A	T
Control (118)	N (%)	30 (25.42%)	73 (61.86%)	15 (12.71%)	133 (56.35%)	103 (43.64%)
Cataract cases (130)	N (%)	66 (50.76%)	51 (39.23%)	13 (10%)	183 (70.38%)	77 (29.61%)
OR/95% CI	3.03/(1.76–5.18)	0.40/(0.24–0.66)	0.76/(0.35–1.68)	1.84/(1.27–2.67)	0.54/(0.38–0.79)
p-Value/chi sq	**< 0.0001/16.75**	**0.0004/12.67**	0.500/0.45	**0.001/10.53**	**0.001/10.53**
Power	0.97	0.923	0.549	0.922	0.922

PPARG2						
Group	Alleles & genotype	CC	CG	GG	C	G

Control (118)	N (%)	25 (21.18%)	47 (39.83%)	46 (38.98%)	97 (41.10%)	139 (58.89%)
Cataract cases (130)	N (%)	15 (11.53%)	114 (87.69%)	1 (0.76%)	144 (55.38%)	116 (44.61%)
OR/95% CI	0.49/(0.24–0.97)	10.76/(5.68–20.41)	0.01/(0.002–0.090)	1.79/(1.25–2.54)	0.56/(0.39–0.80)
p-Value/chi sq	**0.039/4.26**	**< 0.0001/62.22**	**< 0.0001/58.81**	**0.001/10.10**	**0.001/10.10**
Power	0.765	0.999	0.999	0.919	0.919
